# On-Chip OPA: Progress and Prospects in Liquid Crystal, Lithium Niobate, and Silicon Material Platforms

**DOI:** 10.3390/nano15171374

**Published:** 2025-09-05

**Authors:** Xiaobin Wang, Junliang Guo, Zixin Yang, Yuqiu Zhang, Jinyong Leng, Qiang Yu, Jian Wu

**Affiliations:** 1 College of Advanced Interdisciplinary Studies, National University of Defense Technology, Changsha 410073, China; wxb_jobs@163.com (X.W.);; 2 Key Laboratory of Semiconductor Display Materials and Chips & *i*-Lab, Suzhou Institute of Nano-Tech and Nano-Bionics, Chinese Academy of Sciences, Suzhou 215123, China; gjl17613378292@163.com; 3 Nanhu Laser Laboratory, National University of Defense Technology, Changsha 410073, China

**Keywords:** optical materials, optical phased arrays, liquid crystals, lithium niobate, silicon photonics, LiDAR, optical communication

## Abstract

Non-mechanical beam steering is required for holographic displays, free-space optical communication, and chip-scale LiDAR. Optical phased arrays (OPAs), which allow for inertia-free, high-speed beam control via electronic phase control, are an important research topic. The present study investigates the primary material platform for on-chip OPAs: Liquid crystal OPAs (LC-OPAs) employ electrically tunable refractive indices for low-voltage operation; lithium niobate OPAs (LN-OPAs) utilize high electro-optic coefficients for high-speed, low-power consumption, and large-bandwidth operation; and silicon-based OPAs (Si-OPAs) apply mature photonic integration to achieve high integration density and GHz-range steering. The paper thoroughly examines OPA basics, recent material-specific advancements, performance benchmarks, outstanding issues, and future prospects.

## 1. Introduction

Beam steering agility is essential for detection and communication systems, especially for light detection and ranging (LiDAR) [1,2,3,4,5,6,7] and free-space optical communication (FSO) [8,9,10,11]. Phased array technology [12] satisfies these requirements through coherent interference of waves from individual radiating elements, enabling inertia-free beam steering [13,14], dynamic beamforming [15,16], and multi-beam generation [17,18,19] by electronically, precisely, and independently modulating the phase and amplitude of each element, all without relying on mechanical beam control mechanisms. Compared to mechanical systems, phased array technology provides improved multi-target tracking [20,21,22,23], anti-jamming robustness, and compact designs [24,25]. Advances in semiconductor integration and signal processing have extended their application in satellite communications, radio astronomy, and 5G/6G networks [26,27,28,29,30,31], eventually establishing itself as an important core technology underpinning modern electronic systems.

Tunable phase shifters are responsible for the main functioning of a phased array. In microwave and millimeter wave systems, accurate electronic phase modulation is accomplished using materials with customized electromagnetic properties. Ferrite phase shifters, with magnetically tunable permeability, provide direct control over phase delay in waveguide or microstrip transmission lines, acting as the basic building blocks for typical phased array architectures [32,33,34]. Semiconductor phase shifters use their inherent electrical tunability to enable dynamic phase control. These solid-state devices modulate junction capacitances using applied bias voltages or toggling between distinct transmission route lengths, allowing for exact phase shift calibration through entirely electronic means [35,36]. Ongoing research has investigated liquid crystal phase shifters as a means to enhance integration density and performance. These devices use field-induced changes in dielectric anisotropy, where molecular reorientation under applied electric fields alters the effective permittivity, allowing for unique phase-shifting processes with high accuracy [37,38,39]. The basic similarity between these various material techniques is their ability to directly and efficiently convert externally applied electrical control signals (voltage or current) into precise and dynamic phase modulation of microwave or millimeter signals. This method of direct phase modulation via electrical control signals is well established in microwave and millimeter frequency bands. To attain higher spatial resolution and detection performance, beam steering systems must use shorter wavelengths and highly directional light sources. Lasers, with their remarkable coherence and collimation capabilities, emerge as the most effective alternative to achieve these stringent requirements.

**Figure 1:**
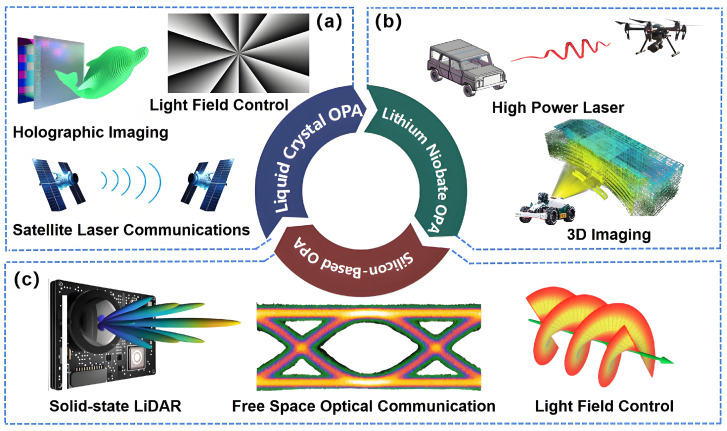
Categories of OPA and related applications.(**a**) LC-OPA for holographic imaging, light field control, and satellite communications; (**b**) LN-OPA for high-power lasers and 3D LiDAR; (**c**) Si-OPA for solid-state LiDAR, free-space optical communication, and light field control.

The optical phased array (OPA) [40,41,42,43] operates on principles analogous to radio-frequency phased arrays. Its core functionality relies critically on a material’s capacity for rapid, reversible, and low-loss optical modulation via external stimuli. Key modulation mechanisms include the electro-optic effect (e.g., in Lithium Niobate, LN) [13,14], the thermo-optic effect (e.g., in Silicon, Si) [44,45], the plasma dispersion effect (e.g., in Indium Phosphide, InP) [46,47], and liquid crystal (LC) birefringence tuning [48,49]. Material-dependent properties, such as electro-optic coefficients, thermo-optic coefficients, and response times, fundamentally dictate OPA performance metrics like beam steering range, ranging capability, and ranging resolution [50]. Consequently, as illustrated in Figure 1, distinct material platforms have naturally found specialized application domains based on these inherent properties.

LC-OPAs exploit tunable birefringence, achieving broadband coverage from visible to infrared wavelengths through scalable element arrays with high resolution. These attributes make them particularly suitable for wearable displays, holographic imaging, light field control, satellite communications, and optical communication systems. LN-OPAs, leveraging high electro-optic coefficients, power handling capabilities, high modulation bandwidth, and high power tolerance, operate primarily in the near-infrared (NIR) spectrum. These characteristics establish them as the preferred technology for demanding applications such as high-power lasers and high-performance 3D LiDAR systems. Si-OPAs capitalize on CMOS compatibility, enabling highly integrated architectures and material versatility. This allows precise engineering of OPA wavelengths across the visible, NIR, and mid-infrared (MIR) spectral ranges. Consequently, Si-OPAs serve as the workhorse for solid-state LiDAR (enabling tiny, low-power systems), free-space optical communication, and light field control.

This paper presents a systematic review of tunable materials for on-chip OPAs. To thoroughly explain the current research environment and the developmental trajectory within this topic, the structure of this review is organized as follows: Starting with fundamental principles, it digs into the main working processes of OPAs, emphasizing crucial performance factors including beam steering, angular resolution (or corresponding beam width), and beam steering range. Second, we systematically categorize and compare the major material systems, such as LC, LN, and Si materials, examining their distinct physical properties and corresponding application scenarios when used in OPA construction, as well as analyzing each material’s inherent advantages and limitations. Finally, based on a study of the existing technological state-of-the-art, this paper analyzes the future problems confronting the OPA area, as well as potential development directions and application opportunities.

## 2. Principle and Characteristics

**Figure 2:**
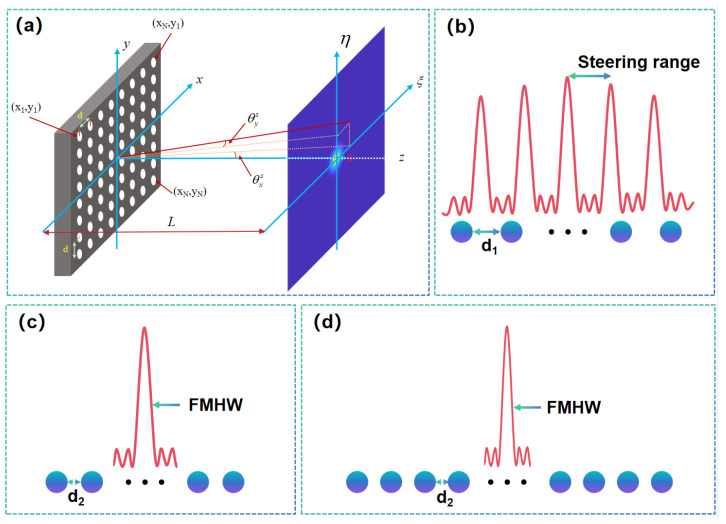
(**a**) Diagram of array beam and its far-field diffraction pattern; (**b**) Multi-beam formation due to element spacing $d_{1}>0.5\lambda$ with annotated steering range; (**c**) Single-beam regime $d_{2}<0.5\lambda$: Beamwidth (FMHW) for baseline array size; (**d**) Beamwidth narrowing with increased element count at fixed $d_{2}<0.5\lambda$.

The far-field radiation pattern of an OPA can be derived from coherent superposition of emitter contributions. For an N×N rectangular array with uniform spacing (Figure 2a), the diffraction plane (x,y) and observation plane (ξ,η) are separated by distance *L*. The center-to-center spacing between array elements is *d*. Assuming each element emits a Gaussian beam with waist diameter $w_{0}$, the position of the (m,n)-th element is $\left(x_{m},y_{n}\right)$ for m,n=1,2,⋯,N. The amplitude of the beam is $a_{m,n}$, and its electric field distribution on the diffraction plane (x,y) is expressed as [51,52]:

(1) $$E_{m,n}\left(x,y\right)=a_{m,n}\exp\left(-\frac{{\left(x-x_{m}\right)}^{2}+{\left(y-y_{n}\right)}^{2}}{w_{0}^{2}}\right)$$

The total electric field distribution in the diffraction plane (x,y) for the N×N OPA is:

(2) $$E_{0}\left(x,y\right)=\sum_{m=1}^{N}\sum_{n=1}^{N}a_{m,n}\exp\left(-\frac{{\left(x-x_{m}\right)}^{2}+{\left(y-y_{n}\right)}^{2}}{w_{0}^{2}}\right)\exp\left(j\phi_{m,n}\right)$$

where $\phi_{m,n}=\phi_{m,n}^{\mathrm{in}}+\phi_{m,n}^{s}$ is the phase of the (m,n)-th element. Here, $\phi_{m,n}^{\mathrm{in}}$ is the initial phase and $\phi_{m,n}^{s}$ is the additional phase required for beam steering to angles $\left(\theta_{x}^{s},\theta_{y}^{s}\right)$:

(3) $$\phi_{m,n}^{s}=\left(m-1\right)kd\sin\theta_{x}^{s}+\left(n-1\right)kd\sin\theta_{y}^{s}$$

where k=2π/λ and λ is the laser wavelength in vacuum.

Based on Fraunhofer diffraction theory [53], the electric field distribution on the observation plane (ξ,η) located at distance *L* from the diffraction plane is given by:

(4) $$\begin{array}{cc} E(\xi ,\eta ) & =\frac{\exp(jkL)}{j\lambda L}\int_{-\infty }^{+\infty }\int_{-\infty }^{+\infty }E_{0}\left(x,y\right)\exp\left[-jk\left(\frac{x\xi +y\eta }{L}\right)\right]dxdy\\ \; & =\frac{\exp(jkL)}{j\lambda L}\pi w_{0}^{2}\exp\left(-k^{2}w_{0}^{2}\frac{\xi^{2}+\eta^{2}}{2L^{2}}\right)\times \sum_{m=1}^{N}\sum_{n=1}^{N}a_{m,n}\exp\left(j\phi_{m,n}\right)\exp\left(-j\frac{k}{L}\left(x_{m}\xi +y_{n}\eta \right)\right) \end{array}$$

The intensity distribution on the observation plane (ξ,η) is given by:

(5) $$\begin{array}{cc} I(\xi ,\eta ) & ={\left|E\left(\xi ,\eta \right)\right|}^{2}\\ \; & =\frac{\pi^{2}w_{0}^{4}}{{\left(\lambda L\right)}^{2}}\exp\left(-k^{2}w_{0}^{2}\frac{\xi^{2}+\eta^{2}}{L^{2}}\right)\times {\left|\sum_{m=1}^{N}\sum_{n=1}^{N}a_{m,n}\exp\left(j\phi_{m,n}\right)\exp\left(-j\frac{k}{L}\left(x\xi +y\eta \right)\right)\right|}^{2}\\ \; & =I_{\mathrm{EN}}\left(\xi ,\eta \right)\times I_{\mathrm{SP}}\left(\xi ,\eta \right) \end{array}$$

where * denotes the complex conjugate operator. Here, $I_{\mathrm{EN}}\left(\xi ,\eta \right)$ is the diffraction factor of a single beam, which determines the envelope of the far-field intensity distribution, and $I_{\mathrm{SP}}\left(\xi ,\eta \right)$ is the interference factor, which determines the coherence characteristics of the far-field intensity distribution:

(6) $$\begin{array}{cc} I_{\mathrm{EN}}\left(\xi ,\eta \right) & =\frac{\pi^{2}w_{0}^{4}}{{\left(\lambda L\right)}^{2}}\exp\left(-k^{2}w_{0}^{2}\frac{\xi^{2}+\eta^{2}}{L^{2}}\right) \end{array}$$

(7) $$\begin{array}{cc} I_{\mathrm{SP}}\left(\xi ,\eta \right) & ={\left|\sum_{m=1}^{N}\sum_{n=1}^{N}a_{m,n}\exp\left(j\phi_{m,n}\right)\exp\left(-j\frac{k}{L}\left(x\xi +y\eta \right)\right)\right|}^{2} \end{array}$$

When the distance *L* between the diffraction plane (x,y) and observation plane (ξ,η) is sufficiently large, the diffraction angles can be approximated as $\theta_{x}\approx \xi /L$ and $\theta_{y}\approx \eta /L$. Equation (5) can be rewritten as:

(8) $$\begin{array}{cc} I(\theta_{x},\theta_{y})= & \frac{\pi^{2}w_{0}^{4}}{{\left(\lambda L\right)}^{2}}\exp\left(-\frac{k^{2}w_{0}^{2}}{2}\left(\theta_{x}^{2}+\theta_{y}^{2}\right)\right)\\ \; & \times \;{\left|\sum_{m=1}^{N}\sum_{n=1}^{N}a_{m,n}\exp\left(j\phi_{m,n}\right)\exp\left(-jk(x\theta_{x}+y\theta_{y})\right)\right|}^{2} \end{array}$$

Under ideal conditions, neglecting phase noise introduced by the system or environment ($\phi_{m,n}^{\mathrm{in}}=0$), and assuming the polarization states remain unchanged during propagation, with all beam amplitudes $a_{m,n}=1$, Equation (8) simplifies to:

(9) $$\begin{array}{cc} I(\xi ,\eta ) & =\frac{\pi^{2}w_{0}^{4}}{{\left(\lambda L\right)}^{2}}{\left[\frac{\sin\left(\frac{1}{2}Nkd\left(\sin\theta_{x}-\sin\theta_{x}^{s}\right)\right)}{\sin\left(\frac{1}{2}kd\left(\sin\theta_{x}-\sin\theta_{x}^{s}\right)\right)}\right]}^{2}\times {\left[\frac{\sin\left(\frac{1}{2}Nkd\left(\sin\theta_{y}-\sin\theta_{y}^{s}\right)\right)}{\sin\left(\frac{1}{2}kd\left(\sin\theta_{y}-\sin\theta_{y}^{s}\right)\right)}\right]}^{2}\\ \; & \;\times \exp\left(-\frac{k^{2}w_{0}^{2}}{2}\left(\theta_{x}^{2}+\theta_{y}^{2}\right)\right) \end{array}$$

where $\left(\theta_{x}^{s},\theta_{y}^{s}\right)$ are the beam steering angles.

### 2.1. Beam Steering

Consider the x-axis component. The maximum intensity occurs when:

(10) $$\frac{\sin\left(\frac{1}{2}Nkd\left(\sin\theta_{x}-\sin\theta_{x}^{s}\right)\right)}{\sin\left(\frac{1}{2}kd\left(\sin\theta_{x}-\sin\theta_{x}^{s}\right)\right)}=1$$

This condition is satisfied when:

(11) $$\sin\theta_{x}-\sin\theta_{x}^{s}=0\;\Rightarrow \;\sin\theta_{x}=\sin\theta_{x}^{s}$$

The relationship between steering angle and phase difference is:

(12) $$\theta_{x}^{s}=\sin^{-1}\left(\frac{\lambda }{2\pi d}\Delta \varphi \right)$$

where Δφ is the phase difference between adjacent array elements.

### 2.2. Beam Steering Range

The main lobe scanning is governed by:

(13) $$\frac{2\pi }{\lambda }d\sin\theta_{M}-\frac{2\pi }{\lambda }d\sin\theta_{x}^{s}=\pm 2i\pi ,\;i=0,\pm 1,\pm 2,\cdots$$

where $\theta_{M}$ is the scanning angle of the beam maximum.

When the main lobe scanning angle $\theta_{x}^{s}=0$:

(14) $$\sin\theta_{M}=\pm \frac{\lambda }{d}i,\;i=0,\pm 1,\pm 2,\cdots$$

Since |sinθ|≤1, grating lobes appear when d≥λ.

When the main lobe scans to its maximum angle $\theta_{\max}$:

(15) $$\sin\theta_{M}=\pm \frac{\lambda }{d}i+\sin\theta_{\max},\;i=0,\pm 1,\pm 2,\cdots$$

To avoid grating lobes at maximum scan angle:

(16) $$d\leq \frac{\lambda }{1+|\sin\theta_{\max}|}$$

According to Equation (16), multi-beam formation occurs when the element spacing exceeds half the wavelength ($d_{1}>0.5\lambda$), as illustrated in Figure 2b, where the annotated steering range highlights the angular coverage. In contrast, a single-beam pattern emerges when $d_{1}<0.5\lambda$ (Figure 2c). This stringent spacing requirement introduces significant fabrication challenges for LiDAR application [54].

### 2.3. Beam Width

The beam width $\theta_{\mathrm{FWHM}}$ is defined as the full width at half maximum (FWHM). According to the sinc function characteristics, the half-power point occurs when:

(17) $$\frac{1}{2}Nkd\left(\sin\theta_{x}-\sin\theta_{x}^{s}\right)\approx 1.39$$

Thus:

(18) $$\sin\theta_{x}-\sin\theta_{x}^{s}=\frac{1.39}{N\pi }\cdot \frac{\lambda }{d}$$

Setting $\theta_{x}=\theta_{x}^{s}+\frac{1}{2}\theta_{\mathrm{FWHM}}$ and using the small-angle approximation, which holds strictly when $\theta_{\mathrm{FWHM}}$ is sufficiently small.

(19) $$\sin\left(\theta_{x}^{s}+\frac{1}{2}\theta_{\mathrm{FWHM}}\right)\approx \sin\theta_{x}^{s}+\frac{1}{2}\theta_{\mathrm{FWHM}}\cos\theta_{x}^{s}$$

Substituting into Equation (18) yields:

(20) $$\theta_{\mathrm{FWHM}}\approx \frac{0.88\lambda }{Nd\cos\theta_{x}^{s}}\;$$

Here, we demonstrate the influence of element count on the full-width at half-maximum (FWHM) of the beam pattern, as calculated using Equation (20). A comparison between Figure 2c,d reveals two antenna arrays with identical element spacing but different aperture sizes. The results clearly show that a larger aperture yields a narrower FWHM, highlighting the direct relationship between array size and angular resolution.

It should be explicitly noted that, due to inconsistencies in parameter terminology across different disciplines and the fact that certain parameters reflect the unique advantages of specific materials, this paper adopts the following unified definitions for key OPA parameters to ensure clarity and precision: beam steering rate refers to the intrinsic response time of the material (denoted as Response time); beam steering range corresponds to the total angular coverage achievable by the beam steering system, i.e., the field-of-view (FOV); and beam width represents the minimum resolvable angular separation between adjacent beams, which is equivalent to the angular resolution.

## 3. Liquid Crystal Optical Phased Arrays

A spatial light modulator (SLM) is a diffractive optical element (DOE) device [37,38,39,55,56]. SLMs have shown great potential in widespread applications, and have been crucial in quantum optics [57,58], microscopy [59], imaging [60,61], optical trapping and tweezers [62,63], materials processing [64], and holography [65]. In this section, we focus on the OPA application of SLM and provide an overview of device scale, deflection angle, response time, and power tolerance capability.

### 3.1. Device Scale

The device scale of an LC-OPA is defined as the number, arrangement, and physical size of independently controllable basic modulation units within it, typically measured in “pixels” or “units”. It is closely related to regulation accuracy, scanning range, and pointing accuracy. In 1989, Raytheon Company in the United States developed the first one-dimensional LC-OPA device, which included 43,000 electrodes. With the help of LC, periodic scintillation gratings were generated to achieve 4 discrete angle deflection, and the highest beam efficiency was 85%. In 1991, Raytheon developed an LC-OPA with an aperture of 4.3 cm × 4.1 cm, which divided 43,000 array elements into 168 sub arrays, and each array’s 256 units could be independently addressed. They implemented control of 43,000 array elements through 256 electrodes. In 1996, Raytheon reported a reflective LC-OPA with an aperture of 2 cm × 2 cm and 5000 elements, which achieved a deflection of 10.6 μm infrared beam within ±5° range, with a diffraction efficiency of 81% at 4.5° [12]. In 2000, BNS Corporation in the United States adopted LC on silicon technology, achieving 8000 quasi continuous deflection angles within ±3° FOV, covering a wavelength range of 633–1550 nm, and an effective optical aperture of 7.4 mm × 6 mm [66]. In 2004, BNS developed an LC-OPA, which had 12,288 pixel units, electrode width of 1 μm, electrode spacing reduced to 0.6 μm, effective aperture increased to 19.66 mm × 19.66 mm, driving voltage increased to 13.2 V, and deflection range at 1550 μm wavelength increased to ±7° [67].

**Figure 3:**
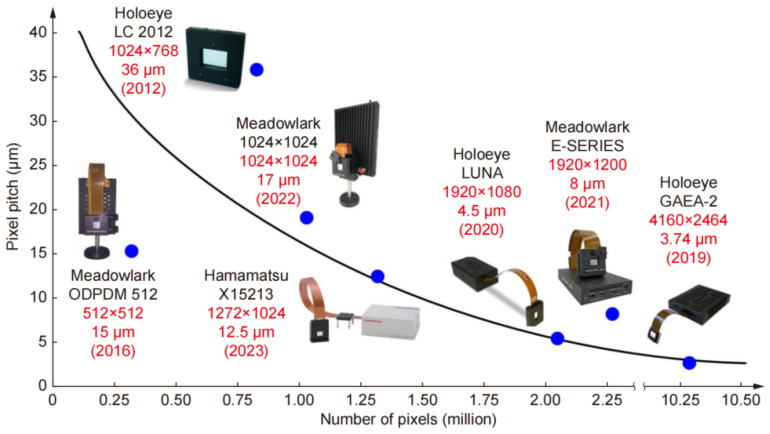
Advances in materials and manufacturing technology have driven significant LC-OPA performance improvements, achieving micrometer-scale pixel pitch with tens of millions of pixels. Reproduced from ref. [68]. Opto-Electronic Journals Group, 2023.

LC-OPAs benefit from semiconductor manufacturing compatibility, enabling scalable mass production. Their strengths in miniaturization and high integration of pixel arrays make them highly promising for aerospace, electronics, and advanced industrial applications. Crucially, as reflected in Figure 3 [68], continuous advances in LC materials and manufacturing technologies have driven significant improvements in LC-OPA performance, characterized by progressively smaller pixel pitch (reduced aperture) and concurrently increasing pixel density (higher number of elements).

### 3.2. Deflection Angle and Diffraction Efficiency

**Figure 4:**
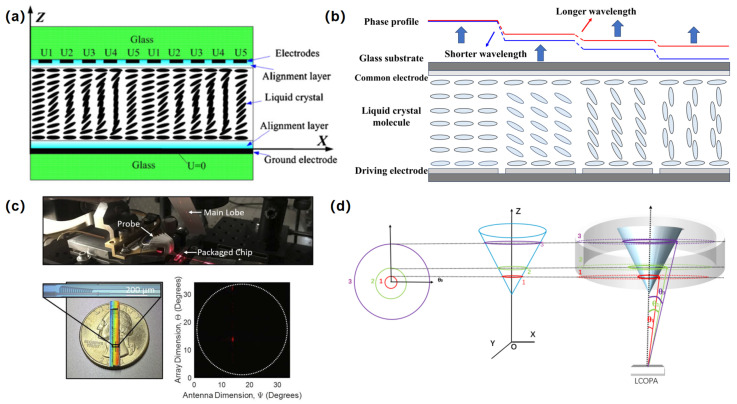
(**a**) Structure profile of LC-OPA. (**b**) Schematic diagram of the phase modulation principle of LC-OPA. (**c**) Photograph of the on-chip LC OPA and the beam steering result. (**d**) Schematic of omnidirectional steering. (**a**) Reproduced from ref. [70]. Copyright©2008 Elsevier Ltd. (**b**) Reproduced from ref. [72]. Optica Publishing Group, 2025. (**c**) Reproduced from ref. [73]. Optica Publishing Group, 2025. (**d**) Reproduced from ref. [74]. Optica Publishing Group, 2025.

The diffraction efficiency of LC-OPAs is defined as the ratio of the diffracted light intensity in a specific direction to the incident light intensity. The diffraction efficiency serves as an indicator that is intimately associated with the device’s inherent space-bandwidth product, material properties, and structural characteristics. BNS successively launched one-dimensional and two-dimensional LC-OPAs. The one-dimensional LC-OPA has 12,288 independently controllable array elements, an LC-OPA aperture of 19.66 mm × 19.66 mm, a zero order light diffraction efficiency of 80–95%, a beam deflection angle range of ±4–7°, an LC response time of 5–30 ms, and a working wavelength range of 635 nm to 1.55 μm. The independent controllable array number of the two-dimensional LC-OPA is 512 × 512, with a working wavelength range of 532 nm to 1.55 μm. In 2008, Jay et al. produced a 1024-element LC-OPA device, which achieved scanning at 60 angles [69]. In 2018, Zhuo Rusheng from the University of Electronic Science and Technology of China proposed a large aperture array design with a PA in PA structure, with a single sub aperture of 10 mm and a total aperture of 100 mm (Figure 4a) [70]. In 2019, He et al. proposed an LC-OPA structure with expandable aperture, which maintained good main lobe focusing performance even when expanded to four times the original optical aperture [71].

In the late 20th and early 21st centuries, Raytheon and BNS in the United States were the first to conduct research on LC-OPAs. Due to technological limitations, the developed devices could only achieve an angle deviation of ±3° to ±7°. In 2008, North Carolina State University successfully developed an infrared LC-OPA that supported ±40° continuous scanning, achieving a large angle diffraction efficiency of over 80% at a wavelength of 1550 nm [75]. In 2012, Wenben et al. achieved the first cascade of LC-OPAs and Wollaston prism, achieving a large angle deflection of ±13.25° [76]. MIT researchers have pioneered an LC-OPA capable of visible-light beam forming and steering, achieving a 7.2° beam-steering range within ±3.4 V driving voltage (Figure 4c) [73]. This advancement represents a critical miniaturization milestone, enabling ultra-compact visible-wavelength systems for applications spanning free-space communications, LiDAR, and holographic displays. The architecture leverages cascaded phase-shifting elements with LC claddings to overcome traditional limitations in modulation efficiency and wavelength compatibility at visible spectra. In 2025, S. L. Zhou et al. revolutionized omnidirectional scanning by integrating an LC-OPA with conical mirrors, achieving 360° × 2.1° continuous FOV and reducing scanning periods to 1/4800 of conventional systems, a pivotal advance for LiDAR applications (Figure 4d) [74].

### 3.3. Response Time

The response time of LC-OPAs refers to the duration required for the device to complete phase modulation switching, encompassing both the rise time (transition from 10% to 90% of maximum phase shift). Such temporal characteristics ultimately determine the achievable beam steering refresh rate and precision in beam control applications. From 2006 to 2007, BNS successively launched one-dimensional and two-dimensional LC-OPAs. The one-dimensional LC-OPA had a response time of 5–30 ms. In 2009, David Engström et al. from the American company Display Technology demonstrated a one-dimensional ferroelectric LC-OPA made of highly tilted ferroelectric LC materials, which could provide 91% phase modulation between 0 and 2π, achieve more than 700 distinguishable deflection angles, and had a response time of less than 200 μs [77]. In 2013, Mengjiao et al. used parallel arranged LC cells as an LC-OPA model for research and obtained the relationship between LC cell thickness and voltage response time [78]. In 2022, He et al. proposed a new three-layer stacked LC-OPA with a deflection angle coverage of ±100 μrad. The emission efficiency in all experiments was higher than 90%, the shortest switching time was only 1.52 ms, and the maximum deflection angle could reach ±260 μrad [79].

### 3.4. Power Tolerance Capability

The power tolerance of LC devices is mainly determined by power density, which is defined as the power value that can be accommodated per unit area. It determines the lifespan and power stability of the equipment. The laser tolerance of LC devices has long been constrained by the limited transmittance of their electrodes within specific wavelength bands. Since the turn of the century, researchers worldwide have conducted extensive research on electrode materials and heat dissipation techniques. For instance, the Transcon 1 device developed by Rockwell and Boeing in 2016 achieved an absorption rate of only 0.2% with active cooling [80]. In 2017, a reflective LC-OPA developed by the University of Electronic Science and Technology of China demonstrated a high-power laser tolerance of 272.4 W/cm^2^. However, even under active cooling its temperature rise reached 14 °C and its reflectivity was limited to 89.87% [81].

**Figure 5:**
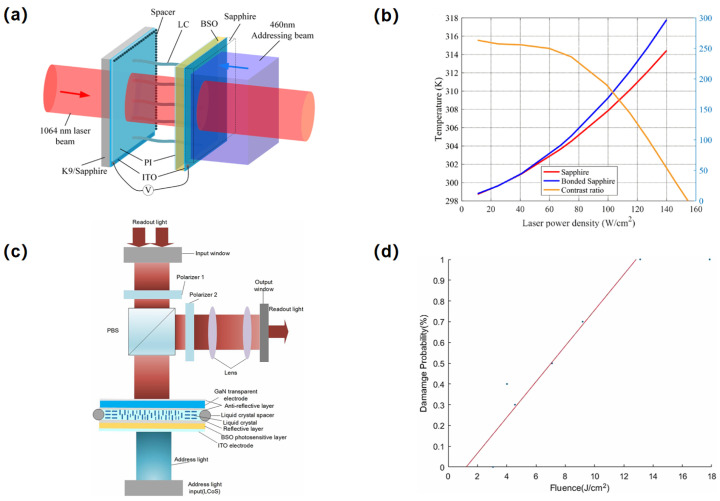
(**a**) SLM structure illustration; (**b**) Temperature of sapphire on both sides of the SLM device as a function of laser power density and the changes in contrast ratio with power intensity; (**c**) The basic structure of the reflective SLM; (**d**) Temperature of sapphire on both sides of the SLM device as a function of laser power density and the changes in contrast ratio with power intensity. (**a**,**b**) Reproduced from ref. [83]. MDPI, 2022. (**c**,**d**) Reproduced from ref. [84]. Cambridge University Press, 2022.

As laser technology advances towards higher energy levels, greater average power, and multi-wavelength applications, the demand for optical field modulation devices, especially LC-OPAs capable of broader wavelength operation, higher repetition rates, and enhanced laser damage thresholds, has grown significantly. To address these challenges, in 2020 Xing et al. [82] developed an LC-OPA employing Si- and Mg-doped GaN as transparent electrodes. Their experiments confirmed that GaN-based LC devices exhibit a high laser damage threshold exceeding 1 J/cm^2^ across both visible and near-infrared regions, demonstrating the potential for high-power GaN-based LC light valves [83] in demanding applications like spatial light modulation for LC-OPAs (Figure 5a,b). Subsequently, in 2022, Du et al. [84] employed laser damage modeling to investigate the damage characteristics of LC devices with ITO versus GaN electrodes under high-power laser irradiation. Analysis of thermal distribution and thermal stress revealed that GaN-electrode LC devices offer superior laser damage resistance and thermal stability compared to ITO-based devices (Figure 5c,d). These advantages are critical for the reliable operation of LC-OPAs in high-power laser systems.

Table 1 shows the performance comparison of the LC-OPA. The development trajectory of LC-OPA technology demonstrates wider steering angles (from ±2° to ±45°) and improved diffraction efficiency (reaching 99.5%). These advancements, coupled with expanding wavelength coverage (450–1550 nm) and recent breakthroughs in high-efficiency designs, indicate the technology is maturing toward practical applications. The ongoing improvements in FOV and response speed are laying essential technical foundations for LC-OPA’s future implementation in real-world systems such as LiDAR and optical communications, where large-angle, high-speed beam steering capabilities are critical requirements.

## 4. Lithium Niobate Optical Phased Arrays

LN [87] has low absorption loss in a wide transparent window. Its Pockels electro-optic (EO) coefficient and second-order nonlinear optical coefficient are relatively high [88]. The electro-optical characteristics of LN endow it with efficient phase modulation capability and high modulation speed [89]. The development of photonic devices on the thin film LN (TFLN) platform has attracted widespread attention from both the industrial and academic communities [90,91,92]. Compared with traditional bulk LN devices, it greatly reduces the footprint and improves performance [93,94]. TFLN modulators have demonstrated pure phase modulation with CMOS-compatible drive voltages and ultra-high bandwidths [95]. Although their fabrication is not yet fully established, this platform still enables the breakthrough potential for achieving systematic, large-scale, and multifunctional photonic chips on a single material platform [96,97,98].

### 4.1. Separate Lithium Niobate OPA

The application of OPA based on LN can be divided into two paths. One is to use bulk LN to form discrete phase modulation devices. Another approach is to use TFLN to form integrated on-chip devices. With the gradual maturity of LN electro-optic modulation devices, which have replaced acousto-optic modulation devices, researchers have proposed various phase control methods and achieved the basic prerequisite for active phase control. It is also a period of rapid development of such OPA in coherent synthesis applications.

**Figure 6:**
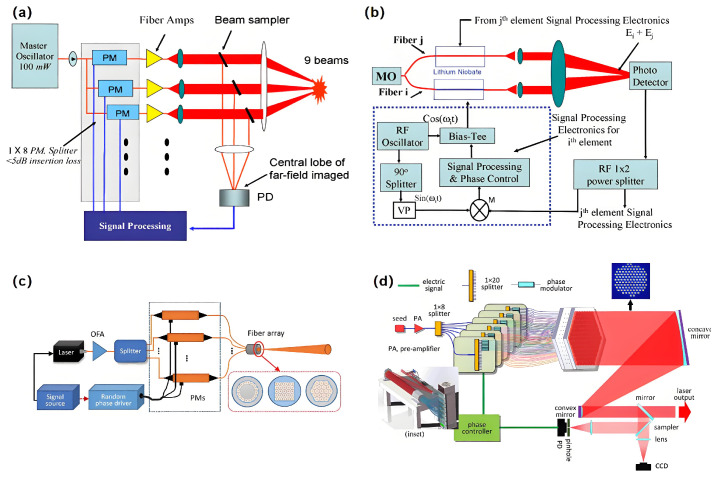
(**a**) Beam combining diagram (PM: phase modulators). (**b**) The electronics based on LN for a 2-element array; (**c**) A computational ghost imaging system using LN phase modulators; (**d**) A High power laser system using LN phase modulators. (**a**,**b**) Reproduced from ref. [100]. Optica Publishing Group, 2006. (**c**) Reproduced from ref. [51]. Optica Publishing Group, 2018. (**d**) Reproduced from ref. [102]. Optica Publishing Group, 2020.

Liu et al. presented the outcomes of phase locking attained through multi-channel spliced fiber optic arrays. Despite the potential for phase distortion in each channel to reach 180 Hz, constructive interference can be attained through the phase locking of the fiber array [99]. T. M. Shay et al. presented an electronic phase-locked optical array utilizing an LN electro-optic modulator platform, independent of an external reference beam. The phase locking of a passive fiber 3 × 3 array and a 6-element fiber amplifier array was achieved, as shown in Figure 6a,b. These optical phase-locked technologies are simple and can resist mechanical vibrations and thermal disturbances. Finally, using these techniques to achieve phase locking in a 22 W fiber amplifier [100], Xiao et al. demonstrated coherent beam combining using 2- and 3-element fiber amplifier arrays in the MOPA configuration. An LN phase modulator with a 500 MHz 3 dB bandwidth was employed in the system [101]. In 2018, Xidian University demonstrated a computational ghost imaging system using an optical fiber phased array employing LN phase modulators, as illustrated in Figure 6c [51]. In 2020, H. C. Chang et al. presented a 107-element coherent fiber array systems using LN phase modulators to modulate phase for the first time (Figure 6d) [102].

### 4.2. Thin Film Lithium Niobate OPA

**Figure 7:**
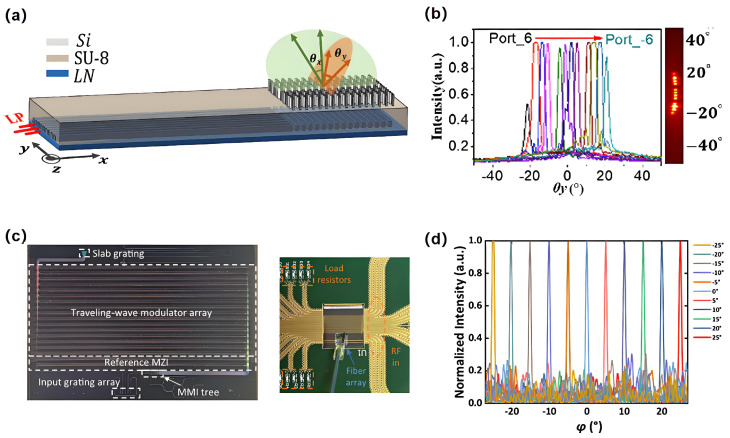
(**a**) Design of the OPA based on LN platform. (**b**) Angle dependent optical power in $\theta_{y}$ direction (±21.27° FOV). (**c**) Image of the fabricated chip and electrical and optical packaged chip; (**d**) Measured optical power distribution versus angle in the phase tuning direction. (**a**,**b**) Reproduced from ref. [105]. Optica Publishing Group, 2022. (**c**,**d**) Reproduced from ref. [106]. Optica Publishing Group, 2023.

The current approach to utilizing a single phased array antenna for two-dimensional scanning involves employing a phase shifter array to manage scanning in one dimension, while adjusting the wavelength facilitates scanning in the orthogonal dimension. The predominant platform for phase shifter arrays is TFLN. In 1974, Tien et al. first reported epitaxial TFLN waveguides based on OPA [103]. This article is among the earliest contributions to the field. Yue et al. presented an integrated LN OPA functioning in the near-infrared region. The system exhibits a two-dimensional beam steering range of 24° × 8°, a full width at half maximum of the far-field beam point measuring 2° × 0.6°, and a sidelobe suppression level of 10 dB [104]. Expanding the FOV of OPA fundamentally depends on the efficient reduction of crosstalk among array elements. In 2022, Tao Li et al. proposed a port-selective OPA based on a metasurface-enhanced LN integrated platform. It provided new degrees of freedom for attaining non-aliasing beam scanning and enhancing the FOV. The metasurface layer above the OPA was engineered to enhance the wide steering range. Consequently, they performed beam scanning throughout an FOV of 41.04° × 7.06° (Figure 7a,b) [105]. In 2023, Zhejiang University considered an on-chip 16-channel OPA based on TFLN phase modulators with traveling-wave electrodes. The OPA achieved an FOV of 50° × 8.6° and a beam width of 0.73° × 2.8° in the phase tuning direction and the wavelength scanning direction, respectively (Figure 7c,d) [106]. Moreover, OPA utilizing sparse array technology to theoretically disrupt the periodicity of waveguide array spacing, resulting in the high-order grating lobes in the far field no longer conforming to the constructive interference condition, thereby achieving the objective of broadening the beam scanning FOV. Shi et al. conceived and tested a two-dimensional scanning OPA utilizing a TFLN phase modulator and a traveling wave electrode. The modulation bandwidth was around 2.5 GHz. A mix of non-periodic arrays and flat grating antennas is employed to mitigate the grating lobes of far-field beams, resulting in an extensive FOV and narrow beam width. A 16-channel OPA has an FOV of 50° × 8.6° and a beam width of 0.73° × 2.8° in the phase tuning and wavelength scanning directions, respectively [106]. Li et al. examined the LN-OPA from a design standpoint. The OPA, consisting of various waveguide arrays, has been adjusted to reduce sidelobes to below −10 dB at a wavelength of 1.55 μm. Simultaneously, OPAs equipped with grating emitters or supplementary silicon dioxide cavities enhance the quality of beam deflection. Subsequently, the one-dimensional beam steering at a 50° angle, governed by an LN-OPA, was demonstrated [107].

**Figure 8:**
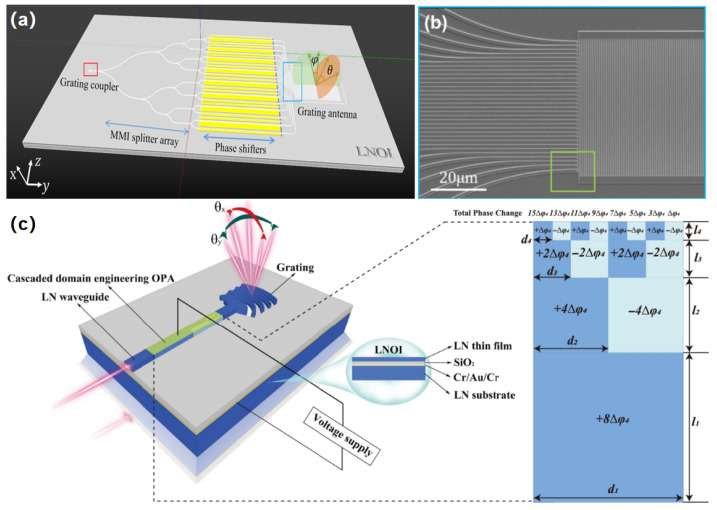
(**a**) Schematic illustration of the OPA chip employing LNOI for two-dimensional beam steering. (**b**) Scanning electron microscopy image of the 32-channel lithium niobate waveguide array. (**c**) Schematic illustration of the four-layer cascaded domain-engineered LNOI-based OPA. (**a**,**b**) Reproduced from ref. [108]. De Gruyter Brill, 2024. (**c**) Reproduced from ref. [110]. Copyright©2025 Elsevier Ltd.

The FOV in wavelength modulation is primarily constrained by the tunable wavelength range of the source and the wavelength tuning efficiency of the antenna. The potential for enhancing wavelength tuning efficiency by optimizing the structure of OPA antennas is significant, primarily through mode multiplexing and multi-line techniques. In 2024, Wang et al. presented a multi-waveguide channel integrated OPA based on the TFLN platform, illustrated in Figure 8a,b. Two LN-OPA chips utilize 32 and 48 channel LN-OPA waveguides, respectively, to achieve an FOV of 62.2° × 8.8° and a beam divergence of 2.4° × 1.2° for the 32-channel configuration through electro-optic modulation, while the 48-channel configuration provides an FOV of 40° × 8.8° and a beam divergence of 0.33° × 1.8° [108]. Furthermore, researchers have suggested a theoretical design of cascaded structures aimed at enhancing the performance of OPAs. Li et al. simulated an OPA architecture based on cascaded periodically poled LN sequences and only two control electronics to program the 2D beam-steering trajectory with a range of $\theta_{y}$ = ±20° and $\theta_{z}$ = ±16° [109]. Li et al. designed a multi-layer cascaded domain engineering structure within the LN waveguide, integrated with wavelength tuning, to facilitate two-dimensional beam scanning through a single electrode controlled OPA, as illustrated in Figure 8c. Simulation results indicated a two-dimensional beam steering capability of 42° × 9.2° [110]. Li et al. proposed a cascaded domain engineering OPA structure. A six-layer cascaded domain amplifier model was designed, comprising 32 array elements. A single electronic control device managed all components of the array. It showed a rapid response utilizing LN electro-optic crystals, with beam control speed reaching up to 3 MHz [111].

**Figure 9:**
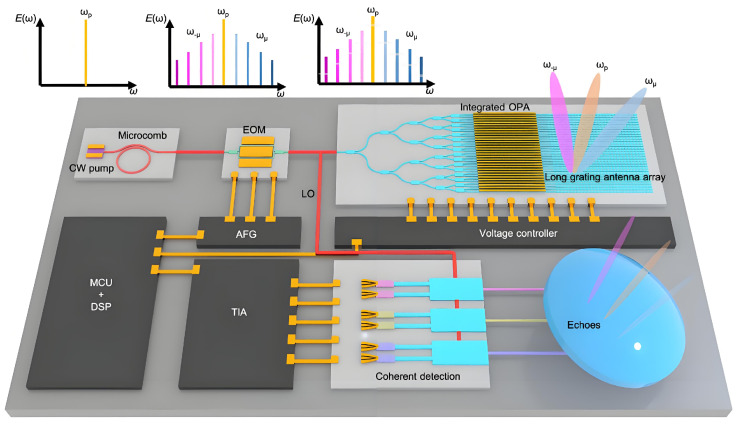
Conceptual illustration of LiDAR system based on integrated TFLN OPA. Reproduced from ref. [112]. Springer Nature Limited, 2025.

The ongoing advancement of OPA technology is anticipated to contribute significantly to the practical implementation of LiDAR. Shi et al. introduced and experimentally validated a hybrid system comprising a silicon integrated OPA and an optical frequency micro comb for parallel laser radar applications, as illustrated in Figure 9 [112]. Each OPA channel can attain 2D steering with an FOV of 80° while avoiding grating lobes in the horizontal direction. Table 2 shows the performance comparison of the LN-OPA.

## 5. Silicon Optical Phased Arrays

**Figure 10:**
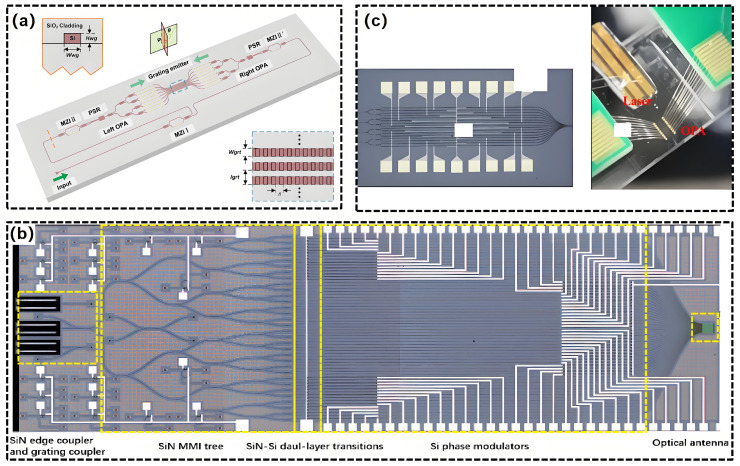
Si materials for OPA. (**a**) SOI. (**b**) SiN. (**c**) Hybrid III/V silicon. (**a**) Reproduced from ref. [115]. IEEE, 2022. (**b**) Reproduced from ref. [120]. Optica Publishing Group, 2020. (**c**) Reproduced from ref. [124]. MDPI, 2024.

The notable difference in refractive indices between silicon and silica facilitates effective optical field confinement, providing a cost-efficient and stable approach for phase modulation. Beyond CMOS compatibility, silicon enable advanced functionalities like optical frequency combs and quantum light sources. Figure 10 illustrates that the advancement of silicon photonics has led to the emergence of various material platforms for OPA, each offering unique benefits. The silicon-on-insulator (SOI) platform [115] (Figure 10a) is notable for its superior optical confinement and seamless integration with established CMOS processes, facilitating the development of monolithically integrated active devices [116,117,118,119] and low-loss passive components [120,121,122,123]. In addition to SOI, various silicon-based platforms have developed to meet particular requirements. SiN waveguides (Figure 10b) can achieve ultra-low transmission loss. Compared with silicon materials, the nonlinear loss of SiN is significantly reduced, supporting high-power laser operation. Its optical transparency window can cover the visible to near-infrared band, making it an ideal platform for visible light OPA, rendering it suitable for long-distance applications, whereas the hybrid III/V silicon platform (Figure 10c) [124] integrates effective light generation with the processing benefits of silicon. Moreover, the hybrid III/V silicon platform (Figure 10c) [124] integrates effective light generation with the processing benefits of silicon, offering complementary advantages. Advanced methodologies such as the Si-SiN multilayered platform enhance light transmission via heterogeneous integration. The inherent CMOS compatibility of these silicon-based solutions provides OPA devices with low cost, low power consumption and rapid scanning capabilities, facilitating their adoption in solid-state LiDAR applications [125,126,127]. The performance of these systems is fundamentally linked to the unique optical properties of each material, including refractive index, thermal-optic coefficients, and loss characteristics. These properties ultimately determine critical OPA metrics such as array scale, ranging resolution, and beam steering rates.

### 5.1. Si-Based OPA

The evolution of OPA technology has progressed through distinct material platforms. Silicon materials exhibit limitations in electro-optic modulation attributed to their low electro-optic coefficients. Nevertheless, their thermal-optic coefficient, approximately $1.86\times 10^{-4}\;K^{-1}$ at a wavelength of 1.55 μm, enables the potential application of the thermal-optic effect.

**Figure 11:**
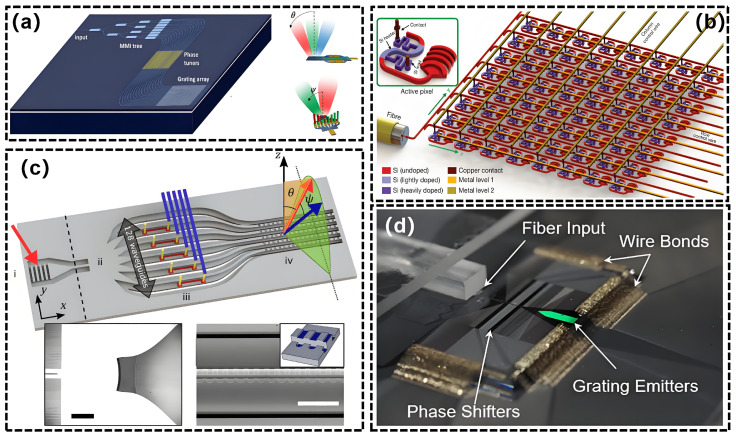
(**a**) A 16-element OPA with independently phase tuned for 2D beam steering. (**b**) A large-scale 64-element 2D OPA. (**c**) A 1D OPA with aperiodic high-resolution. (**d**) Photo of a 512-element OPA. (**a**) Reproduced from ref. [128]. Optica Publishing Group, 2011. (**b**) Reproduced from ref. [116]. Copyright©2013 Springer Nature Limited. (**c**) Reproduced from ref. [130]. Optica Publishing Group, 2016. (**d**) Reproduced from ref. [133]. Optica Publishing Group, 2020.

In 2009, Ghent University demonstrated a one-dimensional 16-element OPA utilizing thermal-optic phase shifters, achieving a beam scanning range of 14.1° × 2.3° [125]. In 2011, a study was conducted on a 16-channel, independently tuned waveguide surface grating OPA in silicon for two dimensional beam steering with a total FOV of 20° × 14° and beam width of 0.6° × 1.6° (Figure 11a) [128]. In 2010, a two-dimensional OPA was proposed, capable of steering beams in both the θ and ϕ directions, utilizing delay lines and grating couplers [127]. The Massachusetts Institute of Technology (MIT) advanced the development of silicon-based OPA for high-density integration and system-level applications. In 2013, researchers presented a large-scale two-dimensional nanophotonic phased array that integrated 64 × 64 optical nanoantennas on a silicon chip. The compact system, with dimensions of 576 μm × 576 μm, successfully demonstrated dynamic beam steering and shaping capabilities through an 8 × 8 sub-array configuration (Figure 11b) [116]. In 2015, the University of Southern California presented the initial demonstration of monolithically integrated 2D OPA featuring photodetectors on a single SOI platform. This study combined the driving circuit with an 8×8 two-dimensional OPA on a single SOI wafer, where each array element was fitted with a thermo-optic phase shifter and attenuator. This configuration facilitated arbitrary beamforming through independently adjustable amplitude and phase [129]. In 2016, Intel technologies introduced a non-uniform one-dimensional 128-element periodic Si-OPA. This study mitigated the formation of grating lobes in the phased array by disrupting the periodicity of the antenna array, enabling beam steering across a 100° × 17° FOV via thermo-optic phase shifting and wavelength tuning (Figure 11c) [130]. In 2017, MIT developed a prototype of a solid-state OPA LiDAR that integrated beam transmission and reception functions. The integration of heterodyne coherent detection technology enabled a 2 m range with a 50° FOV, signifying the practical application of OPA technology in exploration [117]. The team consistently achieved significant advancements and engineering-level upgrades from 2017 to 2020. An OPA system with 512 dimensions incorporated a custom-designed ASIC driver chip, resulting in an FOV of 56° × 15°, a beam width of 0.04° × 0.02°, and a ranging distance of 200 m [131,132]. In 2020, the Columbia University demonstrated a low-power, 2D, 512-element large-scale OPA enabled by a novel multi-pass phase shifter structure [133] (Figure 11d). This structure achieved an FOV of 70° × 6° and consumed power of 1.9 W. In 2020, researchers presented the highest-performance 8192-dimensional ultra-large-scale one-dimensional array, utilizing flip-chip CMOS driver technology. This array features a radiation aperture of 8 × 5 mm^2^, enabling a two-dimensional scan of 100° × 17° and achieving a beam accuracy of 0.01° × 0.039°. This represents a significant industrial advancement in scale and performance for silicon-based OPAs [119].

### 5.2. SiN-Based OPA

Silicon materials are fundamental for effective OPA on SOI platforms, attributed to their elevated refractive index, robust optical confinement, and compatibility with fabrication processes. Additionally, device performance can be improved through the incorporation of low-loss silicon nitride (SiN) materials. Since 2017, SiN has developed into a novel material platform for OPAs due to its low-loss characteristics. MIT has pioneered a CMOS-compatible 4 × 4 mm^2^ SiN phased array operating in the visible spectrum, achieving a significant milestone with 400 mW high-power handling and a beam width of 0.021°. However, the thermo-optic modulation efficiency remains constrained by the material properties [131].

**Figure 12:**
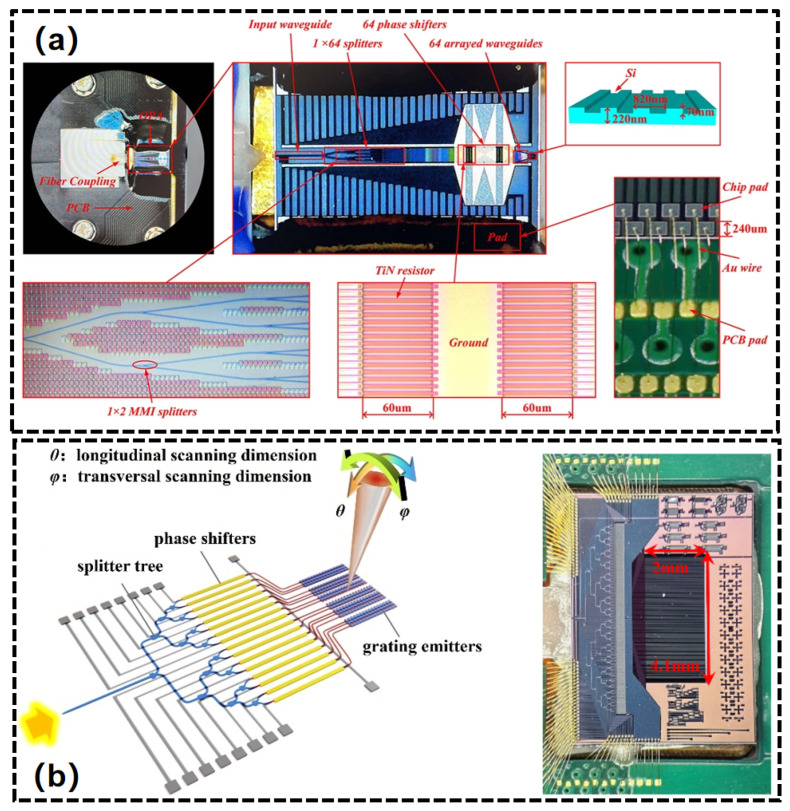
Schematic illustration of a Si-SiN OPA for (**a**) optical communication; (**b**) FMCW LiDAR. (**a**) Reproduced from ref. [137]. Optica Publishing Group, 2024. (**b**) Reproduced from ref. [138]. Optica Publishing Group, 2025.

In 2018, researchers at MIT proposed a novel architecture known as ‘lens-assisted optical path with wavelength tuning’. This design utilizes Mach–Zehnder interferometer (MZI) switches and on-chip integrated lenses to dynamically select optical paths, marking the first demonstration of 38.8° × 12° two-dimensional beam steering on a SiN platform [121]. The Interuniversity Microelectronics Center (IMEC) has developed a dual-layer OPA chip through a SiN/Si bilayer fabrication process. The implementation of a power distribution network featuring SiN edge couplers and cascaded multimode interference (MMI) enhanced the chip’s power tolerance. Thermo-optic phase modulation was subsequently achieved through low-loss interlayer transition structures on silicon waveguides, followed by etching on SiN waveguides to create weakly perturbed dual-layer radiating antenna gratings for off-chip light emission [134]. In 2021, the Shanghai Institute of Microsystem and Information Technology created a 64-dimensional OPA chip utilizing a multilayer SiN/Si process, resulting in an FOV of 35.5° × 22.7° and a beam width of 0.69° × 0.075° [135]. Shanghai Jiao Tong University has reported a LiDAR transmitter that integrates a hybrid tunable external cavity laser with a high-resolution two-dimensional OPA beam steering system. This transmitter, constructed on a three-layer SiN/Si platform, features a widely tunable (100 nm) narrow-linewidth (2.8 kHz) external cavity laser and a 256-dimensional non-uniform OPA with low insertion loss. The chip dimensions are 15.6 mm × 11.7 mm, facilitating an FOV of 140° × 16° and a beam width of 0.051° × 0.016° [136]. The Institute of Semiconductors, Chinese Academy of Sciences, has achieved a breakthrough in passive architecture design by utilizing 27 μm/54 μm delay lines, resulting in scanning densities of 0.44°/nm × 0.07°/nm and 0.87°/nm × 0.07°/nm, respectively. This advancement marks a significant improvement in low phase noise far-field quality. This technical route emphasizes the iterative capabilities of SiN materials in low-power, high-precision OPA systems [123]. In 2024, Jilin University propose a 64-channel Si-SiN dual-layer OPA chip for optical communication applications (Figure 12a) [137]. The system exhibited 6.256 m detection range and 52 Kbps data. In 2025, the Chinese Academy of Sciences presented a 128-channel aperiodic OPA based on a dual-layer emission grating on a SiN-on-SOI platform (Figure 12b) [138]. It achieved an FOV of 150° × 16° with a divergence angle of 0.022° × 0.060°. The OPA was used in a frequency modulation continuous wave system to achieve a distance measurement of 40 m.

### 5.3. Si-SiN-Based OPA

Another important method to increase the output power of the OPA is to input a higher-power laser from the outside [135,136,139,140,141]. Silicon waveguides and devices feature compact dimensions and support diverse functionalities, enabling realization of densely integrated array elements and large-scale OPA chips. However, their performance is limited by nonlinear effects including two-photon absorption, which restricts the operational power threshold (with nonlinear loss typically occurring around 100 mW and damage threshold at approximately 2 W). In contrast, SiN materials exhibit superior characteristics of low optical loss and high nonlinear threshold (where weak nonlinear effects are only observable in high-quality-factor resonant structures, and the damage threshold can reach 35 W), while maintaining full CMOS compatibility. Consequently, a silicon–SiN multilayer integration strategy effectively combines the advantages of both material systems, providing an optimal solution for achieving high main lobe power output—in this hybrid approach, passive components such as power splitters are implemented in SiN to minimize loss, while active thermo-optic or electro-optic phase shifting/modulation elements are fabricated in silicon to maintain functionality.

**Figure 13:**
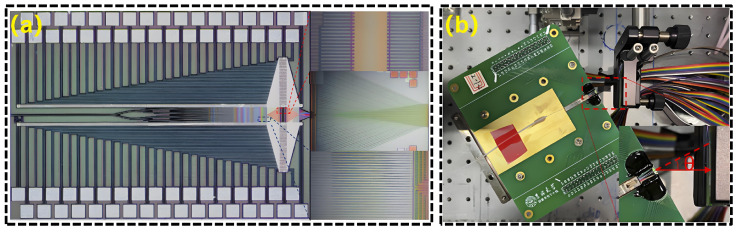
(**a**) Optical microscope picture of Si-SiN OPA chip; (**b**) OPA chip packaging. (**a**,**b**) Reproduced from ref. [146]. Optica Publishing Group, 2021.

The transmitting unit (antenna) may employ a single material or integrate multiple approaches. Interlayer coupling is facilitated by evanescent field coupling [142,143], with coupling loss kept under 0.15 dB [144]. Ref. [145] depicts a large-scale OPA utilizing a multi-layer SiN on silicon photonic platform. The design integrates a hybrid III/V-SiN external cavity laser (ECL) with a 256-channel OPA, resulting in a significant 100 nm wavelength tuning range, a linewidth of 2.8 kHz, and an FOV of 50° × 16° for accurate beam steering [145]. In 2020, the Chinese Academy of Sciences developed a dual-layer OPA utilizing SiN and silicon. This design leverages the low-loss properties of SiN alongside the superior modulation capabilities of silicon, resulting in improved performance of single-layer silicon-based OPA while effectively reducing nonlinear effects associated with high-power optical input in silicon. The system exhibited an FOV measuring 96° × 14° [120]. In 2021, Jilin University developed a 128-dimensional PN junction phase-shifting OPA on the Si-SiN platform, utilizing dual-layer SiN waveguide grating antennas to attain a wide FOV. This configuration achieves a detection range of 100 m when utilized in frequency modulated continuous wave (FMCW) LiDAR systems. As showm in Figure 13 [146].

Table 3 presents a comparison of the performance of various materials utilized in silicon-based OPA. With the maturation of silicon-based OPA technology, commercial OPA LiDAR products are starting to appear on the market. Advancing high-performance OPA systems requires material innovation, specifically through the optimization of thermal management materials on the silicon platform to reduce thermal-optic interference and addressing the material processing challenges linked to III-V/silicon hetero-integration. These advancements will result in compact, low-power, high-speed, and low-loss phased array chips.

## 6. Other Materials for Optical Phased Arrays

Beyond the primary material platforms discussed, several alternative approaches have demonstrated significant potential for OPAs. The early development of AlGaAs-based OPAs laid critical foundations. Hobbs et al. demonstrated a 5-element GaAs OPA, achieving ±20° beam steering at 500 kHz for LiDAR applications. Subsequent advances included a 50-element integrated AlGaAs OPA attaining ±0.41° FOV [147]. In 1997, Xidian University conducted extensive theoretical and experimental work on AlGaAs OPA, leading to the successful fabrication of multiple OPA prototypes [148,149]. However, limited by the low integration capability of AlGaAs materials at the time, this approach failed to advance further. III-V platforms, particularly InP, now enable monolithic integration of active/passive components in the 1.3–1.6 μm band [150], featuring semiconductor optical amplifiers for high-power operation, GHz-speed phase shifters via current injection, and nanosecond-scale tunable lasers. The demonstrated InP OPAs achieve fast beam steering with 10° × 6.5° FOV [151]. III-V/Si hybrid platforms address silicon’s thermal limitations by leveraging quantum-confined Stark/Pockels effects [152,153], achieving breakthrough performance: heterogeneous phase shifters exhibit ultra-low $V_{2\pi }$ voltage (0.35–1.4 V) and minimal residual amplitude modulation (0.1–0.15 dB) across 200 nm [154], while 32-channel arrays with 4μm pitch enable 22° × 28° FOV with 0.78° × 0.02° beamwidth at <3 nW/2*π* shift [153]. Fully integrated 2D OPAs (164 components) achieve 23° × 3.6° FOV on 6 × 11.5 mm^2^ chips [155], and sub-2μm pitch designs demonstrate 51° × 28° FOV [152]. These platforms collectively expand OPA capabilities beyond conventional approaches through high-speed active integration (InP) and voltage-efficient heterogeneous systems (III-V/Si).

## 7. Conclusions and Outlook

This review provides a comprehensive analysis of the progress, advantages, and inherent limitations of OPA technology across three key material platforms: LC, LN, and Si. LC-OPAs exhibit excellent diffraction efficiency and the ability to integrate a large number of phase-shifting elements, enabling high-resolution beam steering and wavefront shaping. These characteristics make them particularly suitable for applications requiring precise optical field manipulation and laser communications. However, their relatively slow response time remains a major constraint for dynamic applications. LN-OPAs offer superior high-speed modulation capabilities (with bandwidths reaching up to 2.5 GHz), low optical loss, and high optical power tolerance, making them ideal candidates for high-power optical communication and quantum optics applications. Nevertheless, challenges remain in improving power efficiency and simplifying the fabrication process. Si-OPAs benefit from mature CMOS compatibility and high integration density, establishing them as the leading platform for photonic integrated circuits and automotive LiDAR systems. However, their application is limited by constraints in optical power handling capacity.

Future progress in OPA technology hinges on addressing platform-specific performance limitations through material innovation. For LC-OPAs, the slow response time represents the most critical limitation for dynamic applications. Therefore, substantial research efforts are required to develop new LC materials, driving schemes, or device architectures capable of achieving microsecond-level or faster switching. For LN-OPAs, despite their excellent modulation speed and power handling capabilities, efforts must focus on reducing fabrication complexity to improve yield and lower production costs. For Si-OPAs, which excel in integration density, further advancements are needed to enhance optical power handling and phase control fidelity and range in order to broaden their applicability in high-power or complex beam manipulation scenarios.

From a performance perspective, as shown in Table 4, the practical deployment of OPAs hinges critically on achieving wide FOV scanning and high-speed beam steering. Realizing a wide FOV fundamentally requires element spacing approaching or even sub-half the operating wavelength (0.5λ) to suppress grating lobes. However, current material platforms face inherent limitations in attaining 0.5λ spacing: LC-OPAs exhibit significant difficulty in achieving sub-wavelength spacing. Nevertheless, techniques such as multi-beam scanning and device cascading have been employed to effectively extend the effective FOV despite this spatial limitation. LN-OPAs, fabricated via semiconductor processing routes, demonstrate potential for reduced element pitch. However, realizing consistent sub-λ spacing demands further breakthroughs in fabrication precision. Similar to LC-OPAs, multi-beam architectures offer a viable pathway to enhance their angular coverage. Si-OPAs, while theoretically capable of 0.5λ designs, practically achieve at best 1.5–2λ spacing in scalable implementations. Aggressive scaling below λ induces severe inter-element crosstalk. This crosstalk degrades phase fidelity across the array, exacerbates sidelobe levels, and ultimately compromises beamforming accuracy.

Recent studies on non-uniform, strongly coupled waveguide arrays, particularly in SiN platforms, demonstrate significant potential for simultaneous control over both optical power distribution and phase profiles across the array [156,157]. This capability substantially expands the functional scope and application potential of OPAs. Additionally, emerging dual-layer platforms that integrate materials such as silicon and SiN offer enhanced flexibility for shaping complex phase profiles [137]. These developments signify a shift toward leveraging innovative photonic designs, moving beyond conventional periodic arrays to achieve functionalities that were previously difficult or unattainable using traditional single-material OPA approaches.

The integration of computational intelligence into OPA design and control presents another promising avenue. Artificial intelligence (AI) algorithms can expedite the optimization of complex antenna layouts, such as non-uniform arrays, improve beam steering accuracy and speed through adaptive control mechanisms, and potentially enable real-time compensation for optical aberrations or environmental disturbances, thereby enhancing system robustness and adaptability.

**Table 4:** The performance comparison of the OPA.

Type	Power Con-Sumption	Footprint	Scale	FOV $\theta_{x}$(°) × $\theta_{y}$(°)	Response Times	Angular Resolution $\theta_{x}$(°) × $\theta_{y}$(°)	Year	Ref.
LC-OPA	–	19.66 mm × 19.66 mm	1 × 12,288	6.95 × –	24.8 ms	–	2006	[69]
	–	80 mm × 80 mm	–	24 × 24	17.2 ms	–	2022	[85]
	–	20 mm × 20 mm	–	22.9 × –	70 μs	–	2022	[158]
	–	15.36 cm × 9.6 cm	1900 × 1200	360 × 2.1	14.0 ms	0.04 × –	2025	[74]
LN-OPA	1.11 nJ/π	48 μm × 100 μm	32	62.2 × 8.8	14 ns	2.4 × 1.2	2024	[108]
	18.5 mW/π	–	128	80 × 5	–	0.1 × 0.037	2025	[112]
	–	–	64	21.22 × –	1.37 μs	0.07 × –	2025	[113]
	41.6 pJ/π	–	16	62 × 7.6	26 ns	3.2 × 1.4	2025	[114]
Si-OPA	8.5 mW/π	576 μm × 576 μm	4096	6 × 6	–	–	2013	[116]
	–	8 mm × 5 mm	8192	100 × 17	30 μs	0.01 × 0.04	2022	[119]
	–	1.8 mm × 2.5 mm	256	14.8 × 150	–	0.066 × 0.068	2023	[141]
	6 mW/π	–	256	160 × –	–	0.16 × 0.04	2024	[159]

While LC-, LN-, and Si-based OPA currently form the technological foundation with distinct advantages for specific applications, the future development of OPA technology will be driven by targeted material and platform optimizations, the potential of hybrid systems, and, critically, the design of novel waveguide architectures leveraging materials like SiN and heterogeneous integration. When combined with intelligent computational control, these advancements are expected to enable unprecedented levels of light manipulation, positioning OPA technology to play a transformative role across a rapidly expanding range of scientific and technological applications.

## Figures and Tables

**Table 1:** Performance comparison of the LC-OPA.

Platform	Scale	FOV $\theta_{x}$(°) × $\theta_{y}$(°)	Response Time (ms)	Angular Resolution $\theta_{x}$(°) × $\theta_{y}$(°)	Wavelength (nm)	Diffractive Efficiency (%)	Year	Ref.
LC	1 × 4096	10 × –	100	–	1550	–	2004	[67]
LC	1 × 12,288	6.95 × –	24.8	–	1550	–	2006	[69]
LC	256 × 256	4 × –	–	–	633	75	2008	[70]
LC	–	24 × 24	17.2	0.001 × 0.001	1550	–	2022	[85]
LC	1900 × 1200	360 × 1.1	–	0.2 × 0.03	632.8	80–91	2025	[86]
LC	1900 × 1200	360 × 2.1	–	0.04 × –	632.8	80–91	2025	[74]

**Table 2:** The performance comparison of the demonstrated (TF) LN-OPA.

Platform	Scale	FOV $\theta_{x}$(°) × $\theta_{y}$(°)	Response Time (ns)	Angular Resolution $\theta_{x}$(°) × $\theta_{y}$(°)	Wavelength (nm)	Bandwidth (GHz)	Year	Ref.
LN	7	0.24 × –	25	0.03 × –	980	10	2014	[13]
LN	64	0.57 × 0.57	–	0.07 × 0.07	1550	0.4	2021	[14]
LN	–	21.22 × –	1376	–	1550	40	2025	[113]
TFLN	16	24 × 8	0.23	2 × 0.6	1550–1600	4.2	2023	[104]
TFLN	16	50 × 8.6	0.4	0.73 × 2.8	1550	2.5	2023	[106]
TFLN	32	62.2 × 8.8	14.4	2.4 × 1.2	1550	–	2024	[108]
TFLN	48	40 × 8.8	14.4	0.33 × 1.8	1550	–	2024	[108]
TFLN	16	62 × 7.6	26	3.2 × 1.4	1550	–	2025	[114]
TFLN	128	80 × 5	–	0.01 × 0.037	1550	3	2025	[112]

**Table 3:** Comparison of the demonstrated Si-OPA.

Platform	Scale	FOV $\theta_{x}$(°) × $\theta_{y}$(°)	Response Time (μs)	Angular Resolution $\theta_{x}$(°) × $\theta_{y}$(°)	Wavelength (nm)	Year	Ref.
Si	16	14.1 × –	–	2.7 × 2.5	1550	2009	[125]
Si	4096	–	–	–	1550	2013	[116]
Si	8192	100 × –	30	0.01 × 0.039	–	2022	[119]
SiN	1024	146 × –	–	0.064 × 0.074	635	2017	[131]
SiN	64	35.5 × 22.7	–	0.69 × 0.075	1550	2021	[135]
SiN	128	150 × 16	–	0.022 × 0.060	1550	2025	[138]
Si-SiN	256	16.1 × 45.6	–	0.044 × 0.154	1550	2022	[140]
Si-SiN	256	14.8 × 150	–	0.068 × 0.066	1550	2023	[141]
